# Multiscale network modeling of oligodendrocytes reveals molecular components of myelin dysregulation in Alzheimer’s disease

**DOI:** 10.1186/s13024-017-0219-3

**Published:** 2017-11-06

**Authors:** Andrew T. McKenzie, Sarah Moyon, Minghui Wang, Igor Katsyv, Won-Min Song, Xianxiao Zhou, Eric B. Dammer, Duc M. Duong, Joshua Aaker, Yongzhong Zhao, Noam Beckmann, Pei Wang, Jun Zhu, James J. Lah, Nicholas T. Seyfried, Allan I. Levey, Pavel Katsel, Vahram Haroutunian, Eric E. Schadt, Brian Popko, Patrizia Casaccia, Bin Zhang

**Affiliations:** 1 Department of Genetics and Genomic Sciences, Icahn School of Medicine at Mount Sinai, One Gustave L. Levy Place, 1470 Madison Avenue, Room S8-111, New York, NY 10029 USA; 2 Icahn Institute of Genomics and Multiscale Biology, Icahn School of Medicine at Mount Sinai, One Gustave L. Levy Place, New York, NY 10029 USA; 3 Medical Scientist Training Program, Icahn School of Medicine at Mount Sinai, One Gustave L. Levy Place, New York, NY 10029 USA; 4 Fishberg Department of Neuroscience and Friedman Brain Institute, Icahn School of Medicine at Mount Sinai, One Gustave L. Levy Place, New York, NY 10029 USA; 5 Department of Human Genetics, Emory University School of Medicine, Atlanta, GA 30322 USA; 6 Department of Biochemistry, Emory University School of Medicine, Atlanta, GA 30322 USA; 7 Integrated Proteomics Core Facility, Emory University School of Medicine, Atlanta, GA 30322 USA; 8 Department of Neurology, The University of Chicago Pritzker School of Medicine, 5841 S. Maryland Avenue, Chicago, IL 60637 USA; 9 Department of Neurology, Emory University School of Medicine, Atlanta, GA 30322 USA; 10 Center for Neurodegenerative Disease, Emory University School of Medicine, Atlanta, GA 30322 USA; 11 Department of Psychiatry, Icahn School of Medicine at Mount Sinai, New York, NY 10029 USA; 12 Mental Illness Research, Education, and Clinical Center (VISN 3), James J. Peters VA Medical Center, Bronx, NY 10468 USA; 13 Neuroscience Initiative, The City University of New York, Advanced Science Research Center, 85 St. Nicholas Terrace, New York, NY 10031 USA

**Keywords:** Alzheimer’s disease, Oligodendrocyte, Myelin, co-expression network, Causal network, RNA sequencing, Proteomics, Differential expression, CNP, BIN1

## Abstract

**Background:**

Oligodendrocytes (OLs) and myelin are critical for normal brain function and have been implicated in neurodegeneration. Several lines of evidence including neuroimaging and neuropathological data suggest that Alzheimer’s disease (AD) may be associated with dysmyelination and a breakdown of OL-axon communication.

**Methods:**

In order to understand this phenomenon on a molecular level, we systematically interrogated OL-enriched gene networks constructed from large-scale genomic, transcriptomic and proteomic data obtained from human AD postmortem brain samples. We then validated these networks using gene expression datasets generated from mice with ablation of major gene expression nodes identified in our AD-dysregulated networks.

**Results:**

The robust OL gene coexpression networks that we identified were highly enriched for genes associated with AD risk variants, such as *BIN1* and demonstrated strong dysregulation in AD. We further corroborated the structure of the corresponding gene causal networks using datasets generated from the brain of mice with ablation of key network drivers, such as *UGT8*, *CNP* and *PLP1*, which were identified from human AD brain data. Further, we found that mice with genetic ablations of *Cnp* mimicked aspects of myelin and mitochondrial gene expression dysregulation seen in brain samples from patients with AD, including decreased protein expression of *BIN1* and *GOT2*.

**Conclusions:**

This study provides a molecular blueprint of the dysregulation of gene expression networks of OL in AD and identifies key OL- and myelination-related genes and networks that are highly associated with AD.

**Electronic supplementary material:**

The online version of this article (10.1186/s13024-017-0219-3) contains supplementary material, which is available to authorized users.

## Background

Alzheimer’s disease (AD) causes a progressive dementia that affects approximately 1/6th of people in the US age 75 and above [1]. Although the number one risk factor for AD is advanced age, the reason for this remains unknown [2, 3]. A wealth of evidence has emerged over the past decade to support the role of non-neuronal cells, especially astrocytes and microglia, in amyloid beta (Aβ) processing and AD pathogenesis [4–6]. While less well studied, several lines of evidence have suggested that dysregulation of oligodendrocytes (OLs) and associated dysmyelination might be important in AD pathology. For example, human neuroimaging studies have shown that white matter changes occur early in AD and are predictive of disease status [7–9]. In particular, MRI studies have detected white matter volume atrophy in multiple brain regions prior to changes in gray matter in AD progression [10–12]. Further, post-mortem human pathological studies have demonstrated that the pattern of neurofibrillary tangle deposition in AD is highly correlated with the developmental pattern of myelination, with late-myelinated axonal tracts substantially more vulnerable to degeneration in AD [13, 14]. Recent reports have highlighted the importance of OLs and myelin metabolic function for axonal health and transport capacity [15–20], thereby suggesting the possibility that OL-driven axonal damage could precede a secondary demyelination in the pathogenesis of AD. In particular, there are several mouse models of ablation of the OL-associated myelin genes *Ugt8* [21], *Cnp* [22, 23], and *Plp1* [24, 25], in which axonal degeneration occurs in the presence of minimal ultrastructural myelin alterations and therefore are well suited to study altered OL gene expression, presumably leading to myeling dysfunction preceding the onset of neurodegeneration. To investigate the hypothesis that OL dysregulation in AD may be part of the underlying mechanism leading to neurodegeneration, we sought to employ a detailed molecular and systems-level analysis to provide a molecular substrate for the potential role of OLs in mediating the initial axonal damage.

In this study, we systematically examined and validated OL-enriched gene networks to uncover key genes and molecular signaling circuits of OLs in AD. We built upon AD-associated and OL-enriched networks constructed in a previous study of genetic, gene expression, and pathophysiologic data in late-onset AD [26]. We constructed a union of the three OL-enriched modules from a multi-tissue AD co-expression network and found that it was strongly enriched for AD risk factor genes. Our OL-enriched consensus module includes genes encoding proteins associated with Aβ-production *PSEN1* and *BACE1,* as well as the AD risk factor genes *BIN1*, *PSEN1*, *PICALM*, and *UNC5C* [27–30]. We next built co-expression networks from a large-scale proteomics data set, identifying a strong loss of coordination among proteins in the most OL-enriched network, an interaction of this dysregulation and dementia status, as well as a down-regulation of key OL network genes, including *BIN1*. We then used the OL modules to construct regulatory networks and found that the topological structures of our OL-enriched networks were validated through in vitro and in vivo perturbations of the predicted key regulatory genes in the networks. Further, transcriptomic analysis of brain tissue isolated from mice with a genetic ablation of three top key driver genes (*Ugt8*, *Cnp*, and *Plp1*) recapitulated key aspects of the dysregulation in gene pathways related to myelination that are seen in human AD brains. We chose to profile the mice with these genetic ablations at an early stage of development (postnatal day 20), in order to detect alterations in gene pathways occurring during the process of myelination and prior to the onset of widespread axon degeneration in *Cnp*-KO [22] or *Plp1*-KO [24] mice. This approach allowed us to define alterations in OL gene expression occurring during the “pre-symptomatic” or prodromal phase of the disease, as opposed to reactive changes that might be consequent to axonal degeneration. We found that differentially expressed gene (DEG) signature in the *Cnp* knockout (KO) mouse mimicked gene expression changes detected in AD brains at the early stages of the pathology, both at the gene pathway and individual protein level, thereby suggesting that dysregulation of OLs in general and *CNP* in particular may play a key role in driving AD-associated gene expression changes.

## Methods

### Oligodendrocyte network construction from AD data and key driver analysis

We re-analyzed data from the Harvard Brain Tissue Resource Center (HBTRC) consisting of 376 late-onset Alzheimer’s disease brain samples as well as 173 non-demented brain samples, harvested from the cerebellum (CBM), dorsolateral prefrontal cortex (Brodmann area 9; henceforth, PFC), and visual cortex (Brodmann area 7; VC), which has been previously described [26] (GEO: GSE44772). Based upon the HBTRC AD data, we previously built up a multi-tissue weighted gene co-expression network using WGCNA [26, 31]. In order to find the AD OL-specific modules, we tested the enrichment of each module for the gene signatures specifically expressed in OLs. To do this, we employed datasets on cell enrichment and identified the genes most highly expressed in myelinating OLs compared to the non-OL cell types [32]. Three modules, one with probes primarily derived from each of the three investigated brain regions were found to be the most enriched for the OL gene signature. We therefore refer to these as the “region-specific oligodendrocyte co-expression network” modules. We further inferred an interaction network for each OL-enriched and region-specific gene module by integrating gene expression and DNA genotypic data, as previously described [26]. Specifically, relationships between genes in each co-expression module were inferred based on conditional independence tests of gene expression in a Bayesian framework, and the presence of more expression single nucleotide polymorphisms (eSNPs) associated with a gene were used as priors to break Markov equivalence [33]. We refer to each of these networks as the “region-specific OL-enriched Bayesian interaction networks”. For robustness, we combined the genes in the three region-specific co-expression networks into a core OL gene set (COLGS), merged the three brain region-specific Bayesian interaction networks by a set union of directed links into a core OL-enriched Bayesian interaction network (COLBN). The gene symbols from this previous study were updated to current Hugo Gene Nomenclature Committee (HGNC) standards using the R package HGCNhelper (v. 0.3.1), and the two gene symbols in the OL-enriched networks that were still mapped to multiple symbols were mapped to the more common symbol (*MARCH1* and *LPAR1*) for legibility. In order to identify key regulatory genes in this interaction network, we then performed key driver analysis on COLBN [34, 35], which uses network connectivity to infer regulatory importance scores for genes.

### Enrichment of brain co-expression modules in AD GWAS risk factor genes

In order to find the enrichment of COLGS and other co-expression modules in Alzheimer’s risk factor genes, we converted the International Genomics of Alzheimer’s Project (IGAP) GWAS SNP-level data set [28] to gene-level *p*-value calls using VEGAS2 [36], and used the genes with significant association at a nominal *p* < 0.05 for further analysis. We then measured the enrichment of this 543 AD GWAS risk factor gene set in the 62 overall qualifying multiscale AD modules with at least 50 genes using Fisher’s Exact Test (FET).

### Proteomics data analysis from PFC human postmortem brain samples

Grey matter brain samples in 50 mg aliquots were harvested from the prefrontal cortex (PFC; Brodmann Area 10) from the autopsied brains of persons with a wide range of cognitive status at the time of death, ranging from no cognitive impairment to dementia, as well as a wide range of Braak scores, from 0 to 6. Liquid chromatography-tandem mass spectrometry (LC-MS/MS) was used to measure the abundance of peptides in each brain sample, from which a protein-level quantitation was estimated using MaxQuant (v1.5.3.30). We used WGCNA to define modules of proteins in the proteomics data, with a soft-thresholding power coefficient of 3. We next annotated these modules based on their relative FET enrichment in the human homologues of genes specifically expressed in each of the five major mouse brain cell types (see “Estimating brain cell type enrichment”).

Within this OL-enriched module, we performed modular differential connectivity analysis in order to assess the overall difference in between samples classified as non-AD (Braak 0–2) and AD (Braak 5–6), calculated using the mean difference in z-scores option in DGCA [37] (v. 1.0.1), with 10,000 permutation samples to assess significance. We used a Student’s t-test to measure differences in average expression between conditions, using the q value R package [38] to estimate false discovery rates in the context of the multiple hypothesis tests.

### Generating in vivo mouse genetic perturbation signatures

#### Animals

Use of animals in this research was strictly compliant with the guidelines set forth by the US Public Health Service in their policy on Humane Care and Use of Laboratory Animals, and in the Guide for the Care and Use of Laboratory Animals. All animal procedures received prior approval from the Institutional Animal Care and Use Committee at Icahn School of Medicine at Mount Sinai.

#### Tissue collection

For each of the control and knockout model mice of the three key drivers (*Cnp*, *Ugt8*, and *Plp1*), mice of either sex were sacrificed at postnatal day 20 and tissue was flash-frozen in liquid nitrogen vapors. The frontal cortex and cerebellum were dissected on ice and immediately processed for RNA isolation. Integrity of the RNA was confirmed by measuring RNA Integrity Number (RIN) and only samples with RIN > 8.5 were used for RNA sequencing.

#### RNA isolation and reverse transcription

RNA was isolated using Trizol reagent (Invitrogen, CA) and cleaned using RNeasy Mini kit (Qiagen, CA). Ribosomal RNA was removed from the samples using Ribo-Zero rRNA Removal Kit (Illumina, CA). Approximately 500 nanograms of total RNA was used in cDNA library construction with the TruSeq RNA Sample Prep Kit (Illumina, CA), followed by RNA sequencing using an Illumina HiSeq2000.

#### Read mapping and quantification of RNAseq data

RNA-sequencing reads were mapped to the mouse genome (mm10, UCSC assembly) using Bowtie (version 2.2.3.0), TopHat (version 2.0.11), and SamTools (version 0.1.19.0) using a read length of 100. For RNAseq knockout experiments, reads were converted to counts at the gene level using HTSeq [39] on the BAM files from TopHat2 using the UCSC known genes data set. For each key driver knockout and brain region, genes that mapped less than 100 counts in 80% or more of the samples were filtered out from downstream analysis, because these genes are likely to have especially high variance in expression calls.

### Compartmental approach to compare of mouse key driver perturbation signatures with human postmortem AD gene expression signatures

We found the enrichment of both mouse key driver knockout DEG signatures, aggregated across significant DEGs (*p* < 0.05, FDR < 0.3) from both the frontal cortex and cerebellum, as well as two human AD gene expression signatures in the gene ontology pathways. The prefrontal cortex (PFC) human AD DEG signature was estimated from the HBTRC cohort. We used the list of genes identified as previously identified as having significantly different RNA levels in brain samples from persons with high levels of Alzheimer’s neuropathology (Braak = 5–6) compared brain samples from persons with low levels of Alzheimer’s neuropathology (Braak = 0–2) via a Student’s t-test [26]. The second human AD DEG signature is from a previous study that identified genes with a significant trend in RNA expression changes across the severity spectrum of AD (control, incipient, moderate, and severe) in the hippocampus (HIPP) [40]. In this data set, we selected the genes as having an increasing or decreasing trend in expression across AD severity stages and an ANOVA *p*-value <0.05 as the HIPP AD DEG signature. We used the moduleGO function in DGCA [37] (version 1.0.1) to perform gene ontology (GO) enrichment analysis on these DEG sets, which leverages the GOstats (version 2.34) [41] and org.Hs.eg.db GO annotation (version 3.1.2) R packages. We filtered for those GO terms with less than 800 and greater than 100 gene symbols. We adjusted the enrichment *p*-values for all GO terms in each DEG set using the Benjamani-Hochberg method. In order to determine whether there was a similar pattern of dysregulation in the mouse key driver knockout models as in human AD, we next found the degree to which the compartment overlaps intersected, using the R package SuperExactTest (version 0.99.2) [42].

### In vivo validation of gene expression

#### Animals

The *Cnp-cre* knock-in mouse line has been described previously [23]. For the sake of clarity, in this manuscript, *Cnp* ^*+/+*^ mice are called *Cnp*-WT and *Cnp* ^*cre/cre*^ mice are called *Cnp*-KO.

#### Western blot

Mice of either sex were sacrificed at postnatal day 60 and brains were cut into 1 mm coronal sections using a refrigerated brain matrix. Corpus callosum (CC) was dissected from coronal sections on ice using a light microscope. The tissue was then immediately processed for protein extraction. Protein lysates (50 μg) were separated by sodium dodecyl sulfate–polyacrylamide gel electrophoresis (SDS–PAGE) and transferred onto a PolyVinylidene DiFluoride (PVDF) (Millipore, Billerica, MA, USA) membrane using a buffer containing 25 mM Tris base, pH 8.3, 192 mM glycine, 20% (vol/vol) methanol for 1 h at 100 V at 4 °C. Membranes were blocked for 1 h in 10% Milk/0.1% Tween/TBS, then incubated overnight at 4 °C with the primary antibody diluted 1:1000 in 5% BSA/0.02% sodium azide/0.1% Tween/TBS. Antibodies used are: mouse anti-CNPase (Sternberger Inc., SMI-91, 1:5,00), mouse anti-alpha-TUBULIN (Calbiochem, CP06, 1:5000), rabbit anti-BIN1 (Abcam, ab185950, 1:2500), rabbit anti-GOT2 (Abcam, ab171739, 1:2500). After rinsing with 0.1% Tween/TBS, membranes were incubated 2 h at room temperature with the secondary light-chain specific antibody (Jackson Immunoresearch, 1:10,000) in 10% Milk/0.1% Tween/TBS. After rinsing, membranes were incubated with ECL (Amersham) for 3 min and then revealed. Quantification was carried out on three biological and technical replicates per genotype, using ImageJ. Protein expressions were normalized to alpha-TUBULIN expression, then compared to their respective expression levels in *Cnp*-WT samples.

#### Immunohistochemistry

Mice were perfused with 4% paraformaldehyde and post-fixed overnight in the same solution at 4 °C. Tissue samples were then transferred to 70% ethanol, sequentially dehydrated and embedded in paraffin. Four-micrometer sections were cut, deparaffinized and rehydrated. Antigen retrieval was performed by incubating slides in sub-boiling (94 °C) citrate buffer (pH 6.0) for 15 min. Slides were incubated in blocking buffer (20% Normal Goat Serum / 1% BSA / PBS 1X) for 1 h at room temperature and then incubated overnight at 4 °C with the primary antibodies in 1% BSA / PBS 1X. Antibodies used are: mouse anti-OLIG2 (Millipore, MABN50, 1:200), mouse anti-NeuN (Millipore, MAB377, 1:200), mouse anti-GFAP (Sternberger Inc., SMI-22, 1:200), rabbit anti-BIN1 (Abcam, ab185950, 1:200), rabbit anti-GOT2 (Abcam, ab171739, 1:200). After rinsing with Tris-buffer / 2% milk, sections were processed with he appropriate Alexa Fluor conjugated secondary antibodies (1:1000 in 1% BSA / PBS 1X, Invitrogen), washed with Tris-buffer, and mounted using Fluoromount-G with DAPI. All images were acquired using a Zeiss Observer A1 fluorescent microscope or Zeiss LSM780 upright Confocal. Quantification was carried out on two-three sections per mouse and three-four mice for each genotype evaluated using ImageJ.

## Results

### A robust myelination- and oligodendrocyte-enriched gene module is strongly associated with genetic data in late-onset AD

**Fig. 1:**
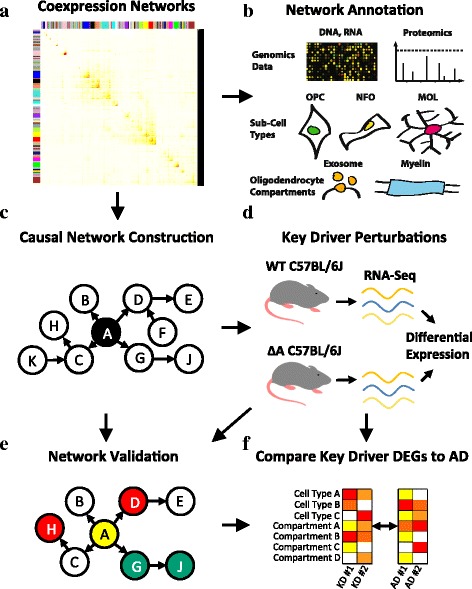
Workflow of the analyses performed in this study. Human postmortem AD brain tissue samples from multiple brain regions were used to construct coexpression networks (**a**) using Weighted Gene Coexpression Network Analysis (WGCNA). The oligodendrocyte/myelination enriched coexpressed gene modules from WGCNA were annotated by a variety of external data sets including DNA, RNA, proteomic, cell type, and proteome compartment data (**b**). Next, Bayesian gene regulatory networks were constructed based on the DNA and RNA postmortem human AD data (**c**). The Bayesian networks were used to identify key driver genes and several key drivers were perturbed in mouse models to identify their downstream targets (**d**). The gene signatures in response to the perturbations of the key driver genes were used to validate the network structure (**e**) and to compare with the differential expression patterns in human AD postmortem brains (**f**)

**Fig. 2:**
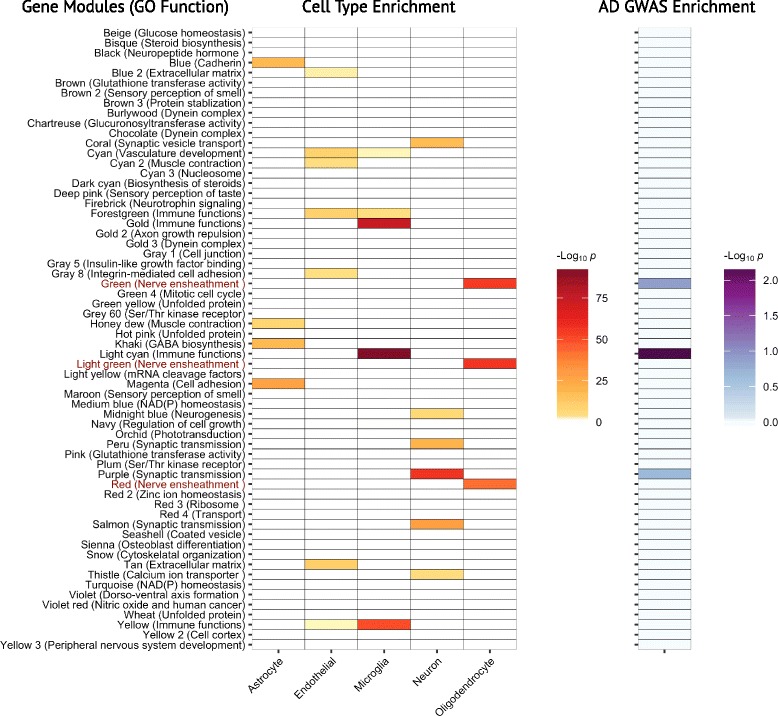
A myelination/oligodendrocyte enriched module in the multi-tissue AD coexpression network is enriched for AD GWAS genes. The left panel shows a heatmap of the enrichments (BH-adjusted -log_10_ *p*-values) of multiscale modules with at least 50 members in marker genes from each of the five major brain cell types, i.e. astrocytes, endothelial cells, microglia, neurons, and myelinating oligodendrocytes, derived from a previous study in mice [32]. The bottom panel shows the enrichment (BH-adjusted -log_10_ p-values) of each corresponding module in the 543 AD risk genes derived from the IGAP AD GWAS study

The primary goal of this study was to interrogate OL-enriched multiscale gene networks constructed from human AD postmortem brain samples (Fig. 1). We first identified co-expressed human gene modules, that were built from data obtained from three regions [e.g. prefrontal cortex (PFC), visual cortex (VC), and cerebellum (CBM)] from postmortem late-onset AD human brains samples from a large cohort of patients [26]. We further annotated these gene modules with additional gene sets to investigate their associations with OLs and AD. We then constructed Bayesian gene regulatory networks for the OL-enriched co-expression modules, identified key regulatory genes in the regulatory networks, and systematically validated the topological structures of the regulatory networks based on a series of gene perturbation experiments in vitro and in vivo. Specifically, in order to identify OL co-expression networks in AD, we tested each of the 62 co-expression modules with at least 50 genes for the enrichment of genes expressed specifically in each of five major brain cell types, i.e., astrocytes, endothelial cells, microglia, neurons, and myelinating OLs [32] (Fig. 2). We identified three co-expression modules with the strongest enrichment of OL genes (Fig. 2), which we subsequently combined, leading to a set of 1631 unique gene symbols, which we henceforth refer to as the core oligodendrocyte gene set (COLGS). Notably, because the three OL-enriched co-expression modules were each primarily identified in one of the three brain regions from the multi-tissue experiment, by combining them we sought to obtain a more sensitive measure of genes whose expression is associated with OLs in the context of AD across brain regions. COLGS is highly enriched for genes encoding proteins identified in the myelin proteome [43] (Fold Enrichment (FE) = 1.92, Fisher’s Exact Test (FET) *p* = 2.4e-15), and is also enriched for genes specifically expressed in each of oligodendrocyte precursor cells (FE = 2.8, *p* = 4.2e-17), newly formed oligodendrocytes (FE = 6.2, *p* = 1.0e-84), and myelinating oligodendrocytes [32] (FE = 7.4, *p* = 2.5e-87), indicating that genes in COLGS capture a wide spectrum of OL functions.

**Fig. 3:**
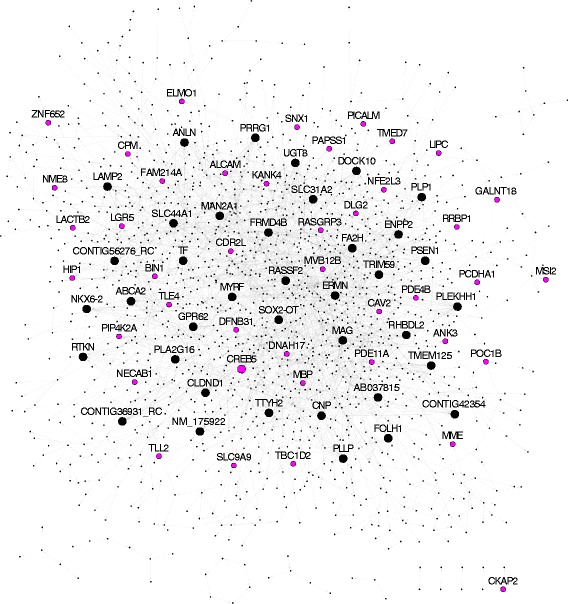
Key drivers and AD GWAS risk genes in the AD oligodendrocyte regulatory network. Visualization of the core oligodendrocyte Bayesian regulatory network (COLBN), where arrows refer to the predicted direction of interaction in the AD sample-derived network. Nodes corresponding to genes that are called as one of the top 40 key drivers in the network are larger sized, while nodes corresponding to genes that are one of the AD GWAS risk factors are colored pink

To systematically evaluate the AD genetics of COLGS, we identified 543 genes with nominally significant (*p* < 0.05) gene-level associations with AD based on a meta-analysis of AD genome-wide association study (GWAS) data by the International Genomics of Alzheimer’s Project (IGAP) [28]. We identified a significant enrichment of the AD risk genes in COLGS (FE = 1.71, *p*-value = 0.0004), due to the presence of *BIN1*, *PICALM*, *NME8*, *SNX1*, and other genes. Of all the individual co-expression modules, one OL-enriched module had the second strongest enrichment for AD GWAS risk genes, second only to an immune- and microglia-enriched module (Fig. 2, Table 1). We further highlighted the specific genes in COLGS that were also identified as AD risk genes (Fig. 3, Table 2). These results suggest that the COLGS contains a relatively large proportion of AD risk genes.

**Table 1:** The region-specific oligodendrocyte-enriched gene networks are among the top associated functional coexpression modules with the 543 significant Alzheimer’s disease GWAS hits

Module Name	Enriched Cell Type	Enriched GO Term	Size	Shared	P-Value	Adjusted P-Value
Light cyan	Microglia	Immune functions	559	23	1.91e-05	0.00118
Green	Oligodendrocyte	Nerve ensheathment	1098	32	0.000389	0.0121
Purple	Neuron	Synaptic transmission	847	26	0.000636	0.0131
Gold	Microglia	Immune functions	400	14	0.00352	0.0546
Salmon	Neuron	Synaptic transmission	750	21	0.00582	0.0722
Burlywood	Ependymal Cell	Dynein complex	114	6	0.008	0.0806
Magenta	Astrocyte	Cell adhesion	841	22	0.0103	0.0806
Gray 1	None	Cell junction	56	4	0.0104	0.0806
Light green	Oligodendrocyte	Nerve ensheathment	474	14	0.0145	0.0998
Seashell	None	Coated vesicle	313	10	0.0222	0.127
Black	None	Neuropeptide hormone	1067	25	0.0226	0.127
Tan	Mural Cell	Extracellular matrix	734	18	0.033	0.171
Blue	Astrocyte	Cadherin	1508	31	0.0557	0.247
Yellow	Microglia	Immune functions	1174	25	0.058	0.247
Honey dew	Astrocyte	Muscle contraction	139	5	0.0613	0.247
Medium blue	None	NAD(P) homeostasis	233	7	0.066	0.247
Red 4	Ependymal Cell	Transport	62	3	0.0682	0.247
Red	Oligodendrocyte	Nerve ensheathment	1089	23	0.0717	0.247
Gold 3	Ependymal Cell	Dynein complex	71	3	0.0936	0.294
Peru	Neuron	Synaptic transmission	408	10	0.095	0.294

The 20 modules with the strongest Fisher’s Exact Test enrichment in AD GWAS genes from the IGAP data set of the 62 multiscale coexpression modules with at least 50 members were considered. P-values were adjusted via the Benjamini-Hochberg method. Size = the number of genes in the module, Shared = the number of overlaps between the IGAP AD GWAS genes and the coexpression modules, GO = Gene Ontology

**Table 2:** The members of the core oligodendrocyte gene set (COLGS) that are identified as significant (p < 0.05) AD GWAS hits in the IGAP data set

Gene	Chromosome	Top SNP	Top SNP *P*-value
BIN1	2	rs35114168	1.05e-25
PICALM	11	rs639012	4.87e-19
NME8	7	rs2060712	6.30e-07
SNX1	15	rs138194169	2.72e-06
CPM	12	rs10878881	1.48e-05
DLG2	11	rs422267	2.09e-05
RASGRP3	2	rs10200743	2.76e-05
GALNT18	11	rs11021857	4.511e-05
DNAH17	17	rs117779187	7.04e-05
LIPC	15	rs17269397	8.32e-05
ZNF652	17	rs12948660	0.000119
MVB12B	9	rs887656	0.000145
CAV2	7	rs75396674	0.000157
CREB5	7	rs42711	0.000158
NFE2L3	7	rs73281529	0.000224
CDR2L	17	rs117639581	0.000299
PIP4K2A	10	rs11013051	0.000390
TLL2	10	rs11594430	0.000542
RRBP1	20	rs6080757	0.000873
KANK4	1	rs114648128	0.00118
SLC9A9	3	rs10804689	0.00141
HIP1	7	rs10259351	0.00165
CKAP2	13	rs58655347	0.00168
TLE4	9	rs62569297	0.00172
NECAB1	8	rs7003020	0.00194
TBC1D2	9	rs73488713	0.00222
LGR5	12	rs75928881	0.00278
PDE4B	1	rs12138629	0.00374
PDE11A	2	rs4893975	0.00380
PAPSS1	4	rs62313402	0.00387
DFNB31	9	rs10817615	0.00450
MBP	18	rs8095585	0.00484
LACTB2	8	rs10097463	0.00545
ALCAM	3	rs114219776	0.00628
TMED7	5	rs10069695	0.00691
CAT	11	rs494024	0.00849
MSI2	17	rs12450585	0.00849
MME	3	rs61758192	0.00885
POC1B	12	rs770369	0.0117
PCDHA1	5	rs2879086	0.0151
ANK3	10	rs117641222	0.0185
FAM214A	15	rs8030871	0.0241
ELMO1	7	rs1420423	0.0333

The p-value column shows the smallest p-value of any associated SNP for each gene

Next, we sought to examine the robustness of COLGS to a variety of sources of variance. We first used data from an independent study that also identified coexpression modules in human AD postmortem brain RNA expression samples [44]. Despite originating from a different brain region (the hippocampus), we found that 83% of the genes in the module with the strongest OL-enrichment in this data set overlapped with the members of COLGS (FE = 18.3, *p* = 9.1e-79). In order to measure the robustness of the co-expression network with respect to an alternative gene expression modality, we utilized a large AD proteomic data set from the prefrontal cortex (PFC; Brodmann area 10), which we corrected for batch, age of death, and sex. We used WGCNA [31] to identify gene modules in this proteomic data set, and found that the module with the strongest OL-enrichment (FE = 1.7, *p* = 0.0053), has 50% overlap with COLGS (FE = 10.7, *p* = 6.6e-58), including the AD risk factor *BIN1* (Additional file 1: Figure S1, Additional file 2). These results suggest a robust co-regulation of the COLGS genes at both the transcript and protein levels in AD.

### Dysregulation of an oligodendrocyte-associated module in AD at the protein level

**Fig. 4:**
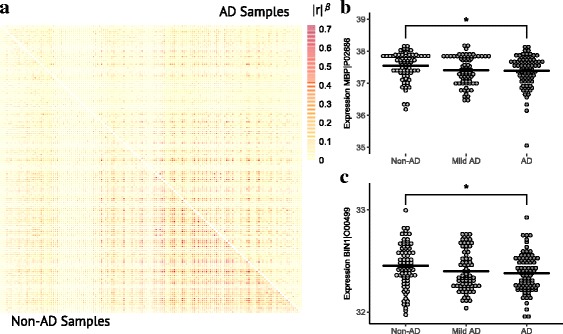
An oligodendrocyte-enriched protein coexpression module is dysregulated in AD. **a** Heatmap of transformed correlations in non-AD samples (Braak <= 2; lower left) and AD samples (Braak > = 5; upper right) in the OL-enriched module consisting of 150 proteins. The transformation consists of taking the absolute value of Pearson correlation coefficients raised to the power of β, i.e., the soft-thresholding power coefficient of 3 used in coexpression network construction. **b**, **c** Expression levels for MBP (**b**) and BIN1 (**c**) in samples classified as non-AD (Braak <= 2), mild AD (Braak 3–4), and AD samples (Braak > = 5). Significance assessed using Student’s t-tests (* = p-value <0.05)

Our previous analysis showed that the three OL-enriched modules were each among the modules with the strongest loss of connectivity in AD samples on the RNA expression level [26]. We sought to extend these results by interrogating gene expression dysregulation in the OL-enriched proteomics module, which includes 150 proteins and is the most OL-enriched module. We found that this module has a significant decrease in correlation in AD samples (mean difference in z-transformed correlation = −0.466, empirical *p*-value = 0.0489; Fig. 4a), thus validating the loss of coordination among OL network genes in AD at the protein level. To further explore gene expression changes in this module in AD, we measured differences in protein expression of the module members, identifying 17 proteins up-regulated in AD and 7 proteins down-regulated in AD at FDR < 0.3 and p-value <0.05 (Additional file 3). Notably, we found that a BIN1 protein isoform was down-regulated in AD (*t* = −2.4, *p* = 0.019, FDR = 0.19), as well as an MBP protein isoform (*t* = −2.3, *p* = 0.023, FDR = 0.19). Therefore, we found that there are both variable changes in expression levels for individual proteins as well as a loss of overall coordination of OL network protein expression in AD.

### Construction and validation of an oligodendrocyte gene regulatory network in AD

We next sought to predict the gene-gene regulatory relationships among the COLGS genes in the RNA expression data, which profiles a wider set of genes than the proteomics data. Specifically, we constructed a Bayesian gene regulatory network for each of the three OL-enriched co-expression modules we identified by integrating RNA expression and genotype data from autopsied brain samples of persons with AD. As detailed in our previous studies [33, 34], Bayesian networks are a type of probabilistic causal network, providing a natural framework for integrating highly dissimilar genetic and gene expression data to predict regulatory relationships. We then combined the three OL Bayesian networks by a set union of directed links, leading to a more robust core oligodendrocyte-enriched Bayesian network (COLBN), and performed key driver analysis on COLBN to identify master regulatory OL genes in the context of AD (Fig. 1b; Table 3).

**Table 3:** Of the 40 top-ranked key drivers from the core oligodendrocyte-enriched Bayesian network (COLBN), 4 have a type of perturbation signature that we used to validate the network, either in vitro or in vivo

Gene Symbol	Major Annotated Roles of Gene Product	Myelin	OL Exosome	Lipid-Binding	AD Risk	Perturbation
ERMN	Myelination	Yes				
UGT8	Galactocerebroside production			Yes		In vivo; this study
CNP	Myelination; Axon interaction	Yes	Yes			In vivo; this study
ENPP2	LPA production					
FRMD4B	?					
PLEKHH1	?					
SLC44A1	Choline transporter	Yes				
TRIM59	?					
PLLP	Myelination	Yes		Yes		
PRRG1	?					
SOX2-OT	lncRNA					
CONTIG36931_RC	?					
CLDND1	?					
ANLN	Actin cytoskeleton	Yes				
TTYH2	Chloride channel					
MYRF	OL differentiation					In vitro; [49]
FA2H	Ceramide hydroxylation			Yes		
CARNS1	Carnosine production					
RNLS	Alpha-NAD(P)H oxidase					
RASSF2	Apoptosis					
PRR18	?					
RTKN	Rho pathway					
DOCK10	Rho pathway					
LPAR1	LPA receptor	Yes		Yes		
MAG	Myelination	Yes				
TF	Iron transport					
TMEM125	?					
PLP1	Myelination; Axon interaction	Yes	Yes	Yes		In vivo; this study
RHBDL2	Protease activity					
NKX6–2	OL differentiation					
FOLH1	Folate hydrolase	Yes				
PSEN1	Protease activity				Yes	
MAN2A1	Glycosylation					
PLA2G16	Phospholipase			Yes		
CONTIG56276_RC	?					
ABCA2	Sterol transport				Yes	
CREB5	?				Yes	
GPR62	?					
SLC31A2	Copper transport					
LAMP2	Glycosylation					

The Major Annotated Roles of Gene Product column is based on a literature review for the major known role(s) of that gene’s product in oligodendrocytes, for which a question mark indicates that no major role is known, to the best of our knowledge. The Myelin column indicates whether or not that gene encodes a protein found in in the myelin proteome [43], the OL Exosome column indicates whether or not that gene encodes a protein found in the OL exosome proteome [97], and the lipid-binding column indicates whether or not that gene encodes a protein found in the set of lipid-binding proteins [98]. The AD risk indicates whether a variant associated with the gene has been previously associated with AD risk, and the Perturbation column indicates whether there is a perturbation signature that we used to corroborate the downstream neighborhood topology for that key driver

**Fig. 5:**
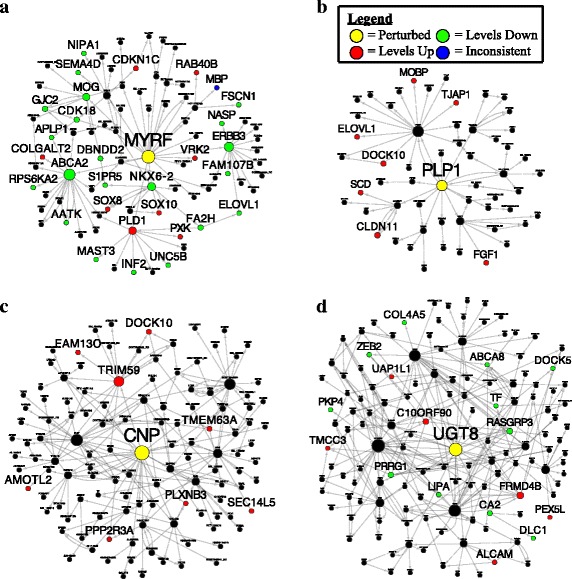
In vitro and in vivo perturbations of key driver genes in the AD myelin/oligodendrocyte networks validated a number of predicted downstream targets. In these network plots, arrows refer to the predicted direction of interaction in the AD sample-derived core oligodendrocyte Bayesian regulatory network (COLBN). The presence of multiple arrows between two genes is because COLBN was constructed by a union of directed links of three networks from three brain regions. In each plot, the perturbed (i.e., knocked-down or knocked-out) gene is colored yellow, the genes significantly down-regulated in the samples with the driver perturbed are colored green, the genes up-regulated are colored red, and the genes with inconsistent expression changes (i.e., multiple probes corresponding to the same gene show opposite directions of changes in expression) are colored blue. The size of the node is proportional to the number of downstream nodes in the subnetwork. **a** Validation of the two-layer subnetwork regulated by *MYRF* using the differentially expressed genes (FDR < 0.3, *p* < 0.05) derived from a *Myrf* knockout experiment in cultured mouse oligodendrocytes. **b**, **c**, **d** Validation of the two-layer subnetworks regulated by *PLP1*, *CNP*, and *UGT8* using the differentially expressed gene signatures (FDR < 0.3, p < 0.05) from the RNAseq data derived from our knockout experiments in mice

**Fig. 6:**
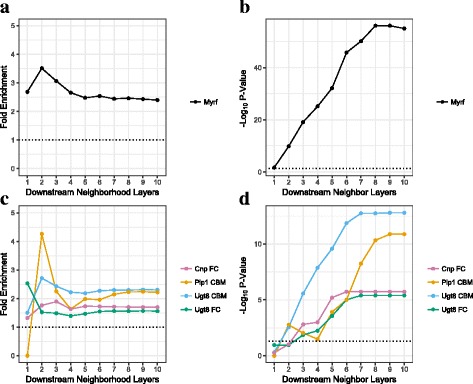
Gene signatures from in vitro and in vivo perturbations of key driver genes in the AD myelin/oligodendrocyte network are significantly enriched in the predicted subnetworks regulated by the driver genes. **a, b** Fold-enrichment (**a**) and BH-adjusted -log_10_ enrichment *p*-values (**b**) for the overlap between the *Myrf* in vitro perturbation signature and each n-layer network neighborhood regulated by *MYRF* in the core oligodendrocyte Bayesian regulatory network (COLBN). **c, d** Fold-enrichment (**c**) and -log_10_ enrichment p-values (**d**) for the overlap between each in vivo perturbation signature and each n-layer network neighborhood regulated by the corresponding driver gene. The result was based on the in vivo knockout differentially expressed gene signatures experiments for *Cnp*, *Plp1*, and *Ugt8* from the cerebellum (CBM) and/or the frontal cortex (FC)

To interrogate the topology of the COLBN as well as how the dysregulation of COLBN key drivers could relate to AD, we identified an in vitro experiment that perturbed a key driver gene in the COLBN, *MYRF*, and examined how the predicted network structures correspond to its identified experimental targets. Specifically, we used data from a previous study that performed transcriptional profiling of cultured mouse OLs with a deletion of the myelination transcription factor *Myrf* (also known as *C11orf9*) [45]. The set of genes differentially expressed in the cells with a *Myrf* deletion compared with the control was significantly enriched in the 5-layer downstream neighborhood of *MYRF* in the COLBN (FE = 2.5, *p* = 7.5e-33; Fig. 5a, Fig. 6a-b), thus validating the topology of the subnetwork regulated by *MYRF*. The validated downstream targets of *MYRF* include *PLD1*, which encodes a protein that regulates the shuttling of *APP* and is associated with *APP* in AD brains [46, 47], as well as *APLP1*, which encodes a protein that has been shown to accumulate within neuritic plaques [48].

Next, we validated the network structure of COLBN in vivo using knockout mouse models of three of the top 40 key drivers in COLBN, *Ugt8*, *Cnp*, and *Plp1*, each of which have been found to cause axon pathology without substantial myelin structural alterations [21, 23, 49]. We performed RNAseq (GEO: GSE80437) on tissue samples of postnatal day 20 mice from the frontal cortex (FC) and cerebellum (CBM) in these three mouse models to determine their KO DEG gene signatures at FDR < 0.3 (Additional files 4 and 5, Additional file 1: Figure S2). We profiled RNA from the FC and CBM because these two regions were also profiled in the human AD postmortem brain tissue study, from which the COLBN was generated. We selected this time point because we wanted to focus on gene changes that occurred prior to the development of axonal pathology and therefore more directly would allow us to determine gene changes characteristic of the prodromic stage of the disease.We identified no DEGs for Δ*Cnp* in the CBM and Δ*Plp1* in the FC, while we found that all of the other DEG signatures were significantly enriched in the downstream neighborhoods of their corresponding driver genes (Fig. 6c-d). *UGT8* encodes an enzyme that transfers galactose to ceramide to generate galactosylceramide, which makes up approximately one-fourth of myelin lipid dry mass [50]. *UGT8*’s 5-layer neighborhood is most enriched for the *Ugt8* KO signature in the CBM (FE = 2.1, *p* = 2.4e-9). One validated downstream target of *UGT8* is *LIPA*, which encodes a lysosomal cholesterol-metabolizing enzyme associated with genetic polymorphisms that affect plasma 24S–hydroxycholesterol/cholesterol levels in AD patients [51] (Fig. 5d). The key driver *CNP* encodes a protein that plays a role in OL process outgrowth and promoting axon survival [23, 52]. *CNP*’s 5-layer neighborhood is significantly enriched for the *Cnp* KO signature in the FC (FE = 1.7, *p* = 6.4e-6). One of the validated downstream targets of *CNP* is *SEC14L5*, which has been previously found to be down-regulated in CA1 in AD [53] (Fig. 5c). Finally, the key driver *PLP1* encodes a protein that is the most abundant protein in CNS myelin sheaths [54], regulates OPC process outgrowth [55], and promotes axonal integrity [56]. *PLP1*’s 5-layer neighborhood is significantly enriched for the *Plp1* KO signature in the CBM (FE = 2.0, *p* = 1.2e-4). A validated downstream target of *Plp1* is *Fgf1* (Fig. 5b), which encodes a protein that has been found to stimulate neuronal growth and promote remyelination [57] and has been found to have increased levels in the CSF in AD [58].

Overall, as with the in vitro perturbation data, the in vivo perturbation experiments strongly support the network structure of COLBN and pinpoint specific downstream targets to explain how dysregulation of the key drivers may help mediate AD pathology.

### Perturbations of key OL network drivers in mice mimic aspects of dysregulation in human AD brain samples

**Fig. 7:**
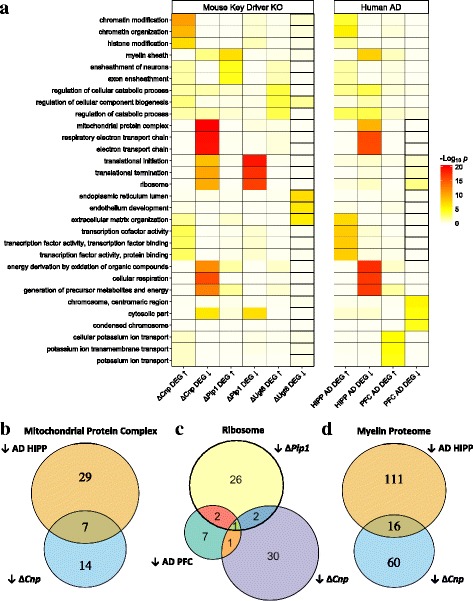
Perturbation of oligodendrocyte network key drivers in mice recapitulates key gene expression changes in human AD brain samples. **a** A heatmap of the -log_10_ gene ontology (GO) enrichment p-values of core oligodendrocyte Bayesian regulatory network key driver knockout differentially expressed gene (DEG) signatures (left panel) and AD DEG signatures from the hippocampus and prefrontal cortex (right panel). The top 3 GO terms with between 100 and 800 gene symbols most enriched in each of the DEG signatures are shown. The p-values for each tested signature were adjusted using the Benjamini-Hochberg method. **b, c, d** Venn diagrams showing the intersections of genes encoding proteins associated with the GO terms “mitochondrial protein complex” (**b**), “ribosome” (c), and genes in the myelin proteome (**d**) with genes downregulated in various DEG signatures are shown

Since the key drivers of COLBN are predicted to orchestrate the expression of a network with a strong genetic association with AD, we hypothesized that the KO signatures of COLBN key drivers in vivo would mimic their gene expression dysregulation in human AD brains. In order to test this hypothesis, we performed gene ontology analysis on DEG signatures from postmortem brain tissue from both key driver KO mice and patients with AD. The DEG signatures in AD were derived based on samples from the PFC in the Harvard Brain Tissue Resource Center cohort [26] and samples from the hippocampus (HIPP) in the University of Kentucky Brain Bank cohort [40], because these regions are among those with the strongest AD pathology. Overall, the enrichment of DEGs from the human AD cases in these gene compartments show several similar dysregulation patterns as that seen in the key driver knockouts (Fig. 7a). For example, we identified a strong enrichment for genes in the gene ontology (GO) category “mitochondrial protein complex” in the down-regulated Δ*Cnp* signature (FE = 10.6, *p* = 8.2e-22) and the down-regulated AD HIPP signature (FE = 6.7, *p* = 7.5e-11). We next found that the down-regulated Δ*Cnp* and AD HIPP signatures intersected along with the mitochondrial gene set substantially more than expected due to chance (FE = 34, *p* = 2.1e-9; Fig. 7b), suggesting that the actual genes dysregulated in the mitochondria are similar between *Cnp*-KO mice and in the hippocampus of AD patients. For example, *COX6A1*, a mitochondrial-associated gene in which mutations are causative of peripheral neuropathy [59], was found to be down-regulated in both *Cnp*-KO mice and in the hippocampus of AD patients. We also identified a strong enrichment for genes in the GO category “ribosome” in the down-regulated Δ*Cnp* signature (FE = 4.8, *p* = 3e-11), the down-regulated Δ*Plp1* signature (FE = 8.1, *p* = 4e-18), and the down-regulated AD PFC signature (FE = 3.0, *p* = 0.002). We identified a significant overlap of all three of these down-regulated signatures and the ribosomal gene set (FE = 76, *p* = 0.013; Fig. 7c), which has limited power due to the small size of the overall ribosomal gene set. Since the *Cnp* and *Plp1* knockout RNAseq profiling occurred prior to the typical age of onset of axon degeneration in these mice, the mitochondrial and ribosomal gene set dysregulation seen is suggestive of “presymptomatic” pathways that precede subsequent axonal and neuronal degeneration.

Finally, we also detected that there was a significant enrichment for genes in myelin sheath GO category in the up-regulated Δ*Plp1* signature (FE = 4.3, p = 3e-8), the down-regulated Δ*Cnp* signature (FE = 2.5, p = 0.002), and the down-regulated AD HIPP signature (FE = 5.2, p = 2e-9). However, the myelin sheath GO signature only contains 161 genes, which makes overlap analysis difficult. In order to use a more well-powered gene set, we utilized a larger set of 1778 genes that have been previously reported to be present in the myelin proteome [43]. In this gene set, we found a strong enrichment for the intersection of genes in the myelin proteome with down-regulated genes in *Cnp*-KO mice and human AD hippocampus (FE = 6.0, p = 2.1e-8; Fig. 7d). This shared set of down-regulated myelin genes includes *CDK5*, which encodes an enzyme whose activity is associated with tau pathology [60] and is essential for myelination [61]. Both *Cnp*-KO mice and patients with AD undergo axon damage in the absence dramatic ultrastructural changes in myelin structure [62], and our data shows that both conditions share a down-regulation of myelin-associated genes that may be associated with dysmyelination [22] and subsequent axon damage.

**Fig. 8:**
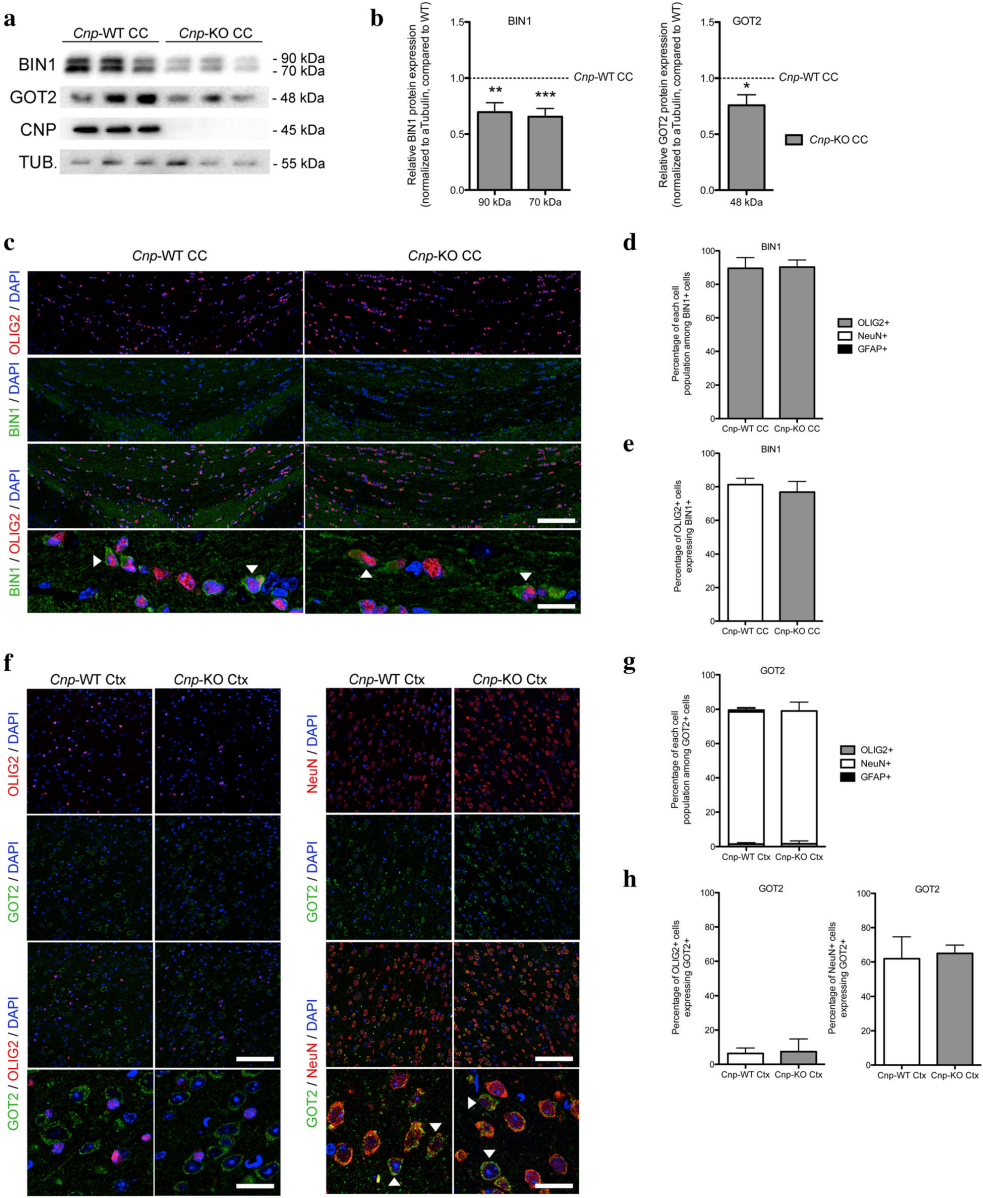
Downregulation of AD-associated proteins in mice lacking the oligodendrocyte network key driver *Cnp*. **a** Representative western blots of BIN1, GOT2, CNP and alpha-TUBULIN stainings on *Cnp*-WT and *Cnp*-KO corpus callosum (CC) tissues (*n* = 3). **b** Quantification of BIN1 and GOT2 protein expression in *Cnp*-KO corpus callosum compared to *Cnp*-WT corpus callosum (protein expression normalized to alpha-TUBULIN expression for each sample, n = 3 per condition; *p* = 0.0041, *p* = 0.0007, *p* = 0.032). **c** Representative confocal image of post-natal day 60 sections of *Cnp*-WT and *Cnp*-KO corpus callosum, stained for OLIG2 (red), BIN1 (green) and DAPI (blue), scale bar = 300 μm. The bottom panel shows higher magnification of the double stainings (white arrowheads indicate double positive for BIN1 and OLIG2), scale bar = 50 μm. **d** Quantification of the proportion of each cell types expressing BIN1 in *Cnp*-WT and *Cnp*-KO corpus callosum (oligodendrocytes – OLIG2+ in gray, neurons – NeuN+ in white, astrocytes – GFAP+ in black; n = 3–4). **e** Quantification of the percentage of OLIG2+ cells expressing BIN1 in *Cnp*-WT and *Cnp*-KO corpus callosum (n = 3–4). **f** Representative confocal image of post-natal day 60 sections of *Cnp*-WT and *Cnp*-KO cortex, stained for OLIG2 or NeuN (red), GOT2 (green) and DAPI (blue), scale bar = 300 μm. The bottom panel shows magnification of the double stainings (white arrowheads indicate double positive for GOT2 and OLIG2 or NeuN, respectively), scale bar = 50 μm. **g** Quantification of the proportion of each cell types expressing GOT2 in *Cnp*-WT and *Cnp*-KO cortex (oligodendrocytes – OLIG2+ in gray, neurons – NeuN+ in white, astrocytes – GFAP+ in black; n = 3–4). **h** Quantification of the percentage of OLIG2+ cells and of NeuN+ cells expressing GOT2 in *Cnp*-WT and *Cnp*-KO corpus callosum (n = 3–4). * p < 0.05, ** *p* < 0.01, *** *p* < 0.001

In order to validate the decreased expression of key myelin and mitochondrial genes in *Cnp*-KO mice, we measured the protein expression of BIN1 and GOT2 in *Cnp*-KO mice compared to WT mice. BIN1 is primarily expressed in mature OLs and white matter in AD patients [63]. Interestingly, the longer, central nervous system (CNS)-specific isoform of *Bin1* has decreased expression levels in AD, although overall *Bin1* transcript levels show increased expression [64]. This may reflect a proliferation of monocyte lineage cells in AD and a concomitant decrease in expression of *Bin1* in other cell types, including OLs. BIN1 protein expression has been found to decrease in the myelin proteome of *Cnp*-KO mice compared to WT mice, but not in *Plp*-KO, *Mag*-KO, or *Sept8*-KO mice [65]. Consistent with this previous data, we found a significant down-regulation of BIN1 expression in the corpus callosum in *Cnp*-KO mice compared to WT mice (*p* = 0.0041 and *p* = 0.0007, Fig. 8a-b). We selected to profile the corpus callosum because it is an OL- and myelin-rich tissue, and therefore we reasoned that protein expression in this region more accurately would reflect the effect of *Cnp*-KO on OLs protein expression. No changes in the subcellular distribution of BIN1 were detected in *Cnp*-KO mice, which was primarily expressed in the cytoplasm of cells that were also stained by antibodies specific for the nuclear pan-OL lineage marker OLIG2 (Fig. 8c-e). Next, we measured the protein expression of GOT2, which is a mitochondrial protein that has been shown to be down-regulated in many AD gene expression studies [66], and has down-regulated transcript levels in *Cnp*-KO mice in our RNAseq expression profiles. Consistent with this data, we found a down-regulation of GOT2 protein levels in *Cnp*-KO mice (*p* = 0.0332; Fig. 8a-b). Although GOT2 has been identified in the myelin proteome, we found that it was primarily co-expressed with neurons (Fig. 8f–h), suggesting that the loss of *Cnp* expression may also affect mitochondrial gene expression within neurons. Taken together, these results validate that *Cnp*-KO mice have gene dysregulation in key myelin and mitochondrial proteins that is similar to that seen in AD brain samples.

## Discussion

In this study, we employed an unbiased systems biology approach to characterize OL-enriched and AD-associated co-expression and regulatory molecular networks. We derived a core OL-enriched gene set (COLGS) that was highly enriched in AD GWAS genes. We found that this set of genes strongly overlapped with the corresponding genes from a protein co-expression module, which we found to be highly dysregulated in AD. We next constructed a core OL Bayesian regulatory network (COLBN) to dissect the causal relationships among the genes in COLGS. By employing a series of in vitro and in vivo perturbations of key driver genes in COLBN, we validated the predicted network structures in COLBN. We further showed that the knockouts of key drivers of COLBN in mice mimic the dysregulation of OL-associated compartments of genes in human postmortem AD brains. In particular, our data revealed a surprisingly strong, convergent gene expression effect of the knockouts *Cnp* and *Plp1* on organelle-associated gene expression pathways, specifically in genes annotated for mitochondrial and ribosome functions. These organelles have also been reported to be dysregulated in axons in AD brains [17, 67–69], suggesting that altered OL-axon communication may lead to dysregulated expression of ribosomal and mitochondrial genes and possibly play a role in contributing to AD axonal pathology. It is difficult to study prodromal changes in late-onset AD brains, because we are not able to determine a priori the individuals who would progress to AD. However, in mice with well-defined ablation of myelin genes also detected as key drivers of human gene network in AD, it is possible to define alterations that occur in OL and that precede frank neurodegeneration. For this reason the detection of similar gene changes in brain tissue samples of *Cnp*-KO and *Plp1*-KO mice prior to the development of any axonal pathology [22, 24], allowed us to infer that the dysregulated expression of similar genes in murine samples and in human AD brains is suggestive of similar events occurring during the early part of the pathological cascade. Overall, this study improves our understanding of the molecular underpinnings of myelination and OLs in AD by identifying biologically relevant pathways, dissecting the causal relationships among the OL- and myelin-related genes, and implicating key driver genes in AD pathogenesis.

At the individual gene level, many of the genes in COLGS have been described as genetic risk factors associated with late-onset AD, including *BIN1* [28], *PICALM* [28], *NME8* [28], *UNC5C* [30], and *PSEN* [27]*.* Notably, *BIN1* is the nearest protein-coding gene to the SNP with the second-strongest GWAS signal for AD, following *APOE* [28]. Histologically, BIN1 is primarily found in the brain at the nodes of Ranvier, consistent with its high RNA expression in OLs and its presence in the myelin proteome [43, 70, 71]. In COLBN, *BIN1* is downstream of *ABCA2*, a cholesterol transporter that has been associated with the risk of AD in many study populations [28, 72–75]. Much of the literature about the role of *BIN1* in AD has focused on its roles in neurons and microglia [76], but our data, in addition to another recent study [63], suggest that its role in OLs should be explored further. In addition to genetic risk factors, our AD OL network also contains many genes encoding proteins that have been associated with AD pathophysiology (e.g.*,* via Aβ production) including *PSEN1* [77], *BACE1* [77], *PLD1* [46, 78], and *APLP1* [79]. Consistent with the important role of *BACE1* in OLs suggested by our network, *BACE1* has been shown to play a key role in myelination [80, 81]. The role of *BACE1* in OLs is of high relevance to AD, as mutations in the *BACE1*-cleaving region of APP have been associated with a decreased risk of AD [82], and β-secretase inhibitors intended to treat AD may have side-effects of myelin defects [83]. A focus on the interaction targets of both *PSEN1* and *BACE1* within OLs using regulatory networks may be a fruitful avenue to identify treatment modalities that decrease deleterious Aβ production without causing off-target effects.

At the gene set level, our enrichment analysis of the AD co-expression modules shows that the three modules significantly enriched for AD risk genes are associated with three different cell types, i.e., microglia, OLs, and neurons (Table 1). Note that gene modules were identified based on the correlation between gene expression profiles in postmortem human AD brains, and they don’t necessarily correspond to any particular known cell types or biological processes due to interactions among cell types and biological processes. Therefore, we denote the co-expression modules by randomly selected color names in addition to their most enriched gene ontology term, to emphasize their multifaceted and highly context-dependent functions. The top ranked module was enriched for immune (microglia/macrophage) genes, consistent with recent reports that immune cells and in particular innate immunity plays a critical role in promoting AD [76, 84–86]. However, it is imprudent to focus on a single cell type and ignore the interactions with other cells. For example, *TREM2*, an established AD risk factor that is primarily expressed in immune cells [87, 88], is also the causative gene of Nasu-Hakola disease, an early-onset subcortical dementia that presents with white matter demyelination [89]. Mice lacking *Trem2* have been shown to have delayed myelin debris clearance, which may lead to increased microglia activation and thus demyelination and neuronal death [90]. The dysregulation of myelin proteome genes that we observed in AD may contribute to pathologic inflammation, by increasing the available lipid pool for scavenging by microglia, which can activate microglia into a pro-inflammatory state [91, 92]. Further investigation of cell type interactions in AD via network biology is a promising approach in addressing the underlying causes of AD.

Existing mouse models of AD tend to focus on Aβ and/or neuronal deficits in AD. For example, several mouse models of AD express genes with familial AD-causative mutations under the *Thy1* promoter [93–95], which is a neuronal marker and will serve to restrict the pathologic changes to neurons. However, the data presented in this study and others suggest that the dysregulation of other cell types, including OLs, may play a role in AD. This opens up a need for mouse models of AD that can recapitulate the OL- and myelin-associated dysregulation in AD. In this study, we found that a mouse knockout model of one of the key drivers in the OL network, *Cnp*, demonstrates a strikingly similar myelin and mitochondrial dysregulation pattern as is seen in brain samples of patients with AD. Notably, we did expression profiling prior to the onset of axon degeneration in *Cnp*-KO mice [22], to minimize the possibility that the myelin gene expression changes observed would be reactive to as opposed to premonitory for axon damage. Taken together, our data suggest that the *Cnp*-KO mice may be a good model of the OL network gene dysregulation and dysmyelination that occurs in the brains of patients with AD. Therefore, therapeutic agents that are able to mitigate and/or prevent the dysmyelination and axon degeneration seen in *Cnp*-KO mice are worthwhile of investigation as potential therapeutic agents for ameliorating cognitive deficits in patients with AD.

In this study, we focused on the molecular networks in AD in a brain cell type, OLs, which have not been widely studied in AD. Our network modeling approach uncovered a network of OL-associated genes that is enriched for AD GWAS genes, pathways through which dysregulation of this OL network may promote AD pathology, and key driver genes that orchestrate these pathways. Further work on our model for the role of OLs in AD may help to address why aging is the major risk factor for AD, since myelin maintenance and plasticity are known to become progressively less robust in normal aging [62, 96]. In particular, preventing or reversing the dysregulation of key OL driver genes such as *CNP* and downstream targets such as *BIN1* deserve further research as interventions to help to alleviate the progression of cognitive deficits in AD.

## Conclusions

This study systematically identifies and validates a comprehensive molecular blueprint of OLs in the context of AD. Our gene network analysis of large-scale genetic and genomic data from AD brains reveals that OL/myelin-enriched subnetworks in multiple brain regions are strongly associated with clinical and neuropathologic traits in AD. These OL/myelin-enriched subnetworks harbor not only Aβ production-related genes, but also several genes involved in myelin biology. Our network analysis further identifies key causal regulators of the OL/myelin-enriched networks, including *UGT8*, *CNP*, *MYRF*, and *PLP1*. These predicted network structures can be validated by gene perturbation signatures of these drivers. Mice with genetic ablations of *Cnp* mimicked components of organelle and myelin sheath gene expression dysregulation seen in brain samples from patients with AD, including decreased protein expression of BIN1 and GOT2. Overall, our network models of OLs in AD and the comprehensive validation experiments reveal details of the molecular mechanisms of OL dysregulation in AD and thus pave a way for the development of novel AD therapies.

## Additional files

Additional file 1: Supplementary Experimental Procedures. **Figure S1.** The topological overlap matrix plot of the protein co-expression network constructed from the proteomics data from the autopsied brains in the MSBB cohort, along with the dendrogram showing the tree cutting process used to define modules (above). **Figure S2.** Confirmation that the key driver knockouts abrogate gene expression of the key driver in the RNA-seq experiments. For each of the key driver knockouts whose genome-wide gene expression was profiled in this study using RNA-seq, we plotted the log_10_ counts overlapping that gene in both the wildtype (WT) and knockout (KO) samples. Notably, one of the matched samples from *Cnp* was detected as an outlier in both the CBM and FC brain regions (red), due to suspected mislabeling. These samples were removed prior to downstream differential expression analysis. (DOCX 161 kb)

Additional file 2: Module membership file for the Mount Sinai Brain Bank (MSBB) proteomic coexpression network. The module label, a randomly chosen color name, is in the 1st column, while the protein name is in the 2nd column. (TSV 34 kb)

Additional file 3: Differentially expressed proteins in the MSBB proteomics data set between AD cases and controls in the OL-enriched module (which has been randomly assigned the color name “Yellow”). (TSV 9 kb)

Additional file 4: Summary of read mapping from the three knockout mouse RNAseq experiments generated by TopHat. (XLSX 52 kb)

Additional files 5: Differentially expressed genes found the mouse knockout RNAseq analyses of *Ugt8* in the CBM (Data 1) and FC (Data 2), *Cnp* in the FC (Data 3), and *Plp1* in the CBM (Data 4). For these differential expression analyses, we mapped RNAseq reads using TopHat, converted to count space using HTSeq, used *voom* to transform the read space data to log_2_ counts per million, and used *limma* for differential expression analysis. We also used the Ensembl database to identify the human gene with the highest homology percentage based on protein coding region DNA divergence, and report this homology percentage for each gene. Note that the differential expression signatures of *Cnp* in the CBM and *Plp1* in the FC were not found not have any differentially expressed genes at FDR < 0.3, so they are not included here. (ZIP 358 kb)

## Abbreviations

AD: Alzheimer’s disease
Aβ: Amyloid beta
CBM: Cerebellum
CNS: Central nervous system
COLBN: Core oligodendrocyte-enriched Bayesian network
COLGS: Core oligodendrocyte gene set
DEG: Differentially expressed gene
eSNPs: Expression single nucleotide polymorphisms
FC: Frontal cortex
FDR: False discovery rate
FE: Fold enrichment
FET: Fisher’s exact test
GEO: Gene expression omnibus
GO: Gene ontology
GWAS: Genome-wide association study
HBTRC: Harvard brain tissue resource center
HGNC: Hugo Gene nomenclature committee
HIPP: Hippocampus
IGAP: International genomics of Alzheimer’s Project
KO: Knockout
MSBB: Mount Sinai Brain Bank
OL: Oligodendrocyte
PFC: Prefrontal cortex
RIN: RNA integrity number
RNAseq: RNA sequencing
VC: Visual cortex
WGCNA: Weighted gene coexpression network analysis
