# Supplementary material for: Multiscale network modeling of oligodendrocytes reveals molecular components of myelin dysregulation in Alzheimer’s disease
Source: Mol Neurodegener. 2017 Nov 6;12:82. doi: 10.1186/s13024-017-0219-3 (PMC5674813; doi:10.1186/s13024-017-0219-3)
Supplement: Supplementary file 1 — Supplementary Experimental Procedures. Figure S1. The topological overlap matrix plot of the protein co-expression network constructed from the proteomics data from the autopsied brains in the MSBB cohort, along with the dendrogram showing the tree cutting process used to define modules (above). Figure S2. Confirmation that the key driver knockouts abrogate gene expression of the key driver in the RNA-seq experiments. For each of the key driver knockouts whose genome-wide gene expression was profiled in this study using RNA-seq, we plotted the log10 counts overlapping that gene in both the wildtype (WT) and knockout (KO) samples. Notably, one of the matched samples from Cnp was detected as an outlier in both the CBM and FC brain regions (red), due to suspected mislabeling. These samples were removed prior to downstream differential expression analysis. (DOCX 161 kb) [file 13024_2017_219_MOESM1_ESM.docx]

**Supplementary Experimental Procedures**

**Co-expression network construction and differential expression analysis**

In the previous analysis of Alzheimer’s disease (AD) and non-demented control data from Harvard Brain Tissue Resource Center [1], mRNA samples were profiled using a custom Agilent 44K array of 39,579 gene-specific DNA probes. In order to limit confounding by demographic variables, tissue sample quality, and batch effects, the data were normalized using robust linear regression for age, sex, postmortem interval, sample pH, and RNA integrity number (RIN). Furthermore, these samples were genotyped for SNPs using the IlluminaHumanHap650Y array and a custom Perlegen 300K array for detecting singleton SNPs. Pathological traits for each AD subject were scored according to the degree of pathology, including Braak score, cortical atrophy, and white matter atrophy. In order to perform coexpression network analysis, briefly, covariance in gene expression patterns across the genome was used to infer relationships between genes. The top 1/3^rd^ most varying probes in each brain region were chosen for co-expression and Bayesian regulatory network analyses to reduce computation complexity. In order to construct the coexpression network in late onset AD (LOAD), a matrix of Pearson correlations between gene expression profiles was computed, followed by a conversion to an adjacency matrix via a power function f(x) = x^β^, where the beta parameter was chosen in order to fit a scale-free adjacency matrix [2]. Differential expression analysis was performed based on a Student’s t-test between LOAD and normal samples in each brain region [1].

**Key driver analysis**

In the combined oligodendrocyte Bayesian interaction network (COLBN), we nominated genes as potential key driver genes based on having an N-hop downstream node number of greater than , where *μ* is the number of N-hop downstream nodes for a particular gene in a particular network [3, 4]. Among the nominated genes, we chose those genes with out-degree of greater than , where d denotes the degree or number of downstream genes, as the key drivers.

**Estimating brain cell type enrichment**

We used a brain cell data set derived from mice that isolated populations of different types of brain cells and performed RNA-seq on them two replicates of the isolated populations each [5]. We downloaded the FPKM values for this data set and log2-transformed them, after adding a constant of 1 to stabilize the log-transformation. For each cell type that we considered, we used limma to measure the genome-wide significance of differences in expression in the cell type of interest compared to the other cell types measured. Further, for each gene, we found the fold-change difference between the cell type of interest and all other cell types. To identify the specifically expressed gene signature of each cell type, we required that each gene have a nominal p-value of < 0.05 and a minimum log fold-change of at least 1.2 in the cell type of interest compared to each of the other cell types considered. For the enrichment analyses of coexpression modules derived from brain tissue, we compared the expression of astrocytes (AST), endothelial cells (END), microglia (MIC), neurons (NEU), and myelinating oligodendrocytes (MOL). For the oligodendrocyte sub-cell type analyses, we compared each of oligodendrocyte precursor cells (OPC), newly formed oligodendrocytes (NFO), and myelinating oligodendrocytes (MOL) to a combination of samples from the other four cell types (AST, END, MIC, NEU).

**Processing of IGAP Alzheimer’s Disease GWAS data**

The International Genomics of Alzheimer's Project (IGAP) is a large two-stage study based upon genome-wide association studies (GWAS) on individuals of European ancestry. In stage 1, IGAP used genotyped and imputed data on 7,055,881 single nucleotide polymorphisms (SNPs) to meta-analyse four previously-published GWAS datasets consisting of 17,008 Alzheimer's disease cases and 37,154 controls (The European Alzheimer's disease Initiative – EADI the Alzheimer Disease Genetics Consortium – ADGC The Cohorts for Heart and Aging Research in Genomic Epidemiology consortium – CHARGE The Genetic and Environmental Risk in AD consortium – GERAD). In stage 2, 11,632 SNPs were genotyped and tested for association in an independent set of 8,572 Alzheimer's disease cases and 11,312 controls. Finally, a meta-analysis was performed combining results from stages 1 & 2. We downloaded the data from the IGAP website (http://web.pasteur-lille.fr/en/recherche/u744/igap/igap_download.php) and used the combined meta-analysis results for further processing. However, the data is at the SNP level, rather than the gene level. In order to address this issue, we used VEGAS2 [6] to convert the combined IGAP table (specifically, the SNP IDs and their associated p-values) to a gene-level description of the association with AD risk. For this procedure, we used all of the SNPs from the 1000 Genomes European data set and a gene definition of 0kb that required the associated SNP to either be within the gene or in linkage disequilibrium of >= 0.8 r^2^ with a SNP within the gene. For downstream analysis, we chose all of the genes that were associated with AD with p-values of less than 0.05.

**Generating proteomics data from autopsied brain samples from the Mount Sinai Brain Bank**

Due to the postmortem nature of this project, it was designated as exempt human research by the Icahn School of Medicine Institutional Review Board.

**Sample Preparation**

Grey matter was dissected from the prefrontal cortex (BM10) of snap-frozen never-thawed specimens and divided into 50 mg aliquots and submitted for proteomic analysis. The specimens were derived from the brains of persons with either no neuropathology or only AD-associated neuropathology (neuritic plaques and neurofibrillary tangles). The studied cohort was relatively evenly divided with respect to cognitive status at the time of death with an average of 38 brains in each of 7 groups comprised of persons with no cognitive deficits (clinical dementia score (CDR)=0), mild cognitive impairment (CDR=0.5), or different severities of dementia ranging from CDR1 to CDR5. Similarly, the studied cohort was comprised of persons with no neuritic plaques in BM10 through persons with more than 20 neuritic plaques per mm^2^. Braak scores ranged from 0 for 12 subjects to an average of 32 subjects for each of Braak scores of 2-6.

**LC-MS/MS analysis**

**Tissue Processing and Batch analysis**

All 266 cases were randomized by traits (e.g. age, sex, PMI, cognitive status and neuropathology) into seven total batches (n = 38 cases each) for homogenization and protein digestion. Pulverized brain tissue was resuspended and homogenized in 500 uL of urea lysis buffer (8M urea, 100 mM NaHPO4 buffer system, pH 8.5), including 5 μL (100x stock) HALT protease and phosphatase inhibitor cocktail (Pierce). All homogenization was performed using a Bullet Blender (Next Advance) according to manufacturer protocols. Protein concentration was determined by BCA. Protein concentration was determined by the bicinchoninic acid (BCA) method, and samples were frozen in aliquots at −80°C. For each batch, protein homogenates (150 ug) were diluted with 50 mM NH_4_HCO_3_ to a final concentration of less than 2 M urea and then treated with 1 mM dithiothreitol (DTT) at 25°C for 30 minutes, followed by 5 mM iodoacetimide (IAA) at 25°C for 30 minutes in the dark. Protein was digested with 1:100 (w/w) lysyl endopeptidase (Wako) at 25°C for 2 hours and further digested overnight with 1:50 (w/w) trypsin (Promega) at 25°C. Resulting peptides were desalted with a Sep-Pak C18 column (Waters) and dried under vacuum.

**LC-MS/MS analysis**

Brain derived tryptic peptides (2 μg) were resuspended in peptide loading buffer (0.1% formic acid, 0.03% trifluoroacetic acid, 1% acetonitrile) and analyzed by LC-MS/MS essentially as previously described [7]. One pooled peptide reference standard comprised all 38 samples in batch 1 were also analyzed in the beginning, middle and end of all seven batches. Peptide mixtures were separated on a self-packed C18 (1.9 um Dr. Maisch, Germany) fused silica column (25 cm x 75 μM internal diameter; New Objective, Woburn, MA) by a NanoAcquity UHPLC (Waters, Milford, FA) and monitored on a Q-Exactive Plus mass spectrometer (ThermoFisher Scientific, San Jose, CA). Elution was performed over a 120-minute gradient at a rate of 400 nL/min with buffer B ranging from 3% to 80% (buffer A: 0.1% formic acid and 5% DMSO in water, buffer B: 0.1 % formic and 5% DMSO in acetonitrile). The mass spectrometer cycle was programmed to collect one full MS scan followed by 10 data dependent MS/MS scans. The MS scans (300-1800 m/z range, 1,000,000 AGC, 150 ms maximum ion time) were collected at a resolution of 70,000 at m/z 200 in profile mode and the MS/MS spectra (2 m/z isolation width, 25% collision energy, 100,000 AGC target, 50 ms maximum ion time) were acquired at a resolution of 17,500 at m/z 200. Dynamic exclusion was set to exclude previous sequenced precursor ions for 30 seconds within a 10 ppm window. Precursor ions with +1, and +6 or higher charge states were excluded from sequencing.

**Label-free protein quantification by MaxQuant**

Data files from each of the seven individual batches and reference standards were analyzed by MaxQuant v1.5.3.30 with Thermo Foundation 2.0 for RAW file reading capability. The search engine Andromeda was used to build and search a concatenated target-decoy UniProt Knowledgebase (UniProtKB) containing both Swiss-Prot and TrEMBL human reference protein sequences (90,411 target sequences downloaded April 21, 2015), plus 245 contaminant proteins included as a parameter for Andromeda search within MaxQuant [8]. Methionine oxidation (+15.9949 Da), asparagine and glutamine deamidation (+0.9840 Da), and protein N-terminal acetylation (+42.0106 Da) were variable modifications (up to 5 allowed per peptide); cysteine was assigned a fixed carbamidomethyl modification (+57.0215 Da). Only fully tryptic peptides were considered with up to 2 miscleavages in the database search. A precursor mass tolerance of ±20 ppm was applied prior to mass accuracy calibration and ±4.5 ppm after internal MaxQuant calibration. Other search settings included a maximum peptide mass of 6,000 Da, a minimum peptide length of 6 residues, 0.05 Da tolerance for high resolution MS/MS scans. The false discovery rate (FDR) for peptide spectral matches, proteins, and site decoy fraction were all set to 1%. The label free quantitation (LFQ) algorithm in MaxQuant (MaxLFQ) [9, 10] was used for protein quantitation. Each batch (i.e, parameters group) was quantified separately by MaxLFQ. The quantitation method only considered razor and unique peptides for protein level quantitation.

***In vitro* oligodendrocyte gene expression perturbation signature**

Data to identify the *Myrf* perturbation signature was downloaded from GEO (GSE15303) after being generated and normalized using an Affymetrix microarray in a previous study that compared gene expression in isolated mouse OLs samples with wildtype genotype (n = 4) to those lacking *Myrf* (n = 3) [11]. This RNA expression data was log-transformed prior to differential expression analysis.

**Calling differential expression gene signatures**

All RNA expression samples were checked for quality, and one outlier sample from the frontal cortex was removed from the *Cnp* RNA-seq experiment due to suspected mislabeling (**Supplementary Figure 2**). In order to use a comparable method for differential expression, the counts from the RNA-seq experiments were converted to normalized matrices using the voom method of the R package *limma* (version 3.24.12). We next used *limma* to make differential expression gene (DEG) calls between the samples with a knockout or knockdown (more generally, perturbed expression) of a key driver gene compared to samples with wildtype levels of expression for that key driver, using an empirical Bayesian approach to model the mean-variance relationship across genes. We adjusted for multiple hypothesis tests by estimating qvalues using the qvalue R package, which accounts for dependence among p-values [12, 13]. To identify DEG signatures from each experiment, we used a false discovery rate (FDR; i.e., q-value) cutoff of 0.3 and a nominal p-value cutoff of 0.05, since we are interested in the relative enrichment of the DEG set, and in targeted validations of predictions from the Bayesian interaction network, as opposed to unsupervised identification of differentially expressed genes. To convert from mouse to human gene names where necessary, we used the Ensembl database (accessed through biomaRt) to convert from mouse to human homologous gene symbols, and used the human gene with the highest homology percentage based on protein coding region DNA divergence in the case of multiple homologous genes.

**Gene enrichment analysis and multiple hypothesis testing adjustments**

The enrichment analyses used in this paper utilized Fisher’s Exact Test for quantifying the significance of the gene set enrichment, equivalent to the hypergeometric test for enrichment. The universe of gene symbols in these enrichment tests was the total number of unique symbols in the data used to generate the target gene set. We used the Benjamini-Hochberg (BH) adjustment method [14] to adjust the Fisher’s Exact Test (FET) enrichment p-values derived from the annotations of the OL-associated signatures in COLGS, the OL-enriched coexpression modules from the independent study and the proteomics data in COLGS, the AD GWAS significant risk genes in the multiscale modules, the perturbation signatures in the downstream neighborhood layers of each perturbed gene, as well as the gene compartments in each of the key driver knockout DEG signature and human postmortem AD DEG signatures.

**Overlap of key driver signatures with downstream members of co-expression networks**

For each gene with a perturbation signature, we identified the N-hop downstream nodes in the core OL-enriched Bayesian interaction network (COLBN). We call the full set of genes that are cumulatively less than N-hops downstream of a particular key driver gene a “layer,” so that the genes in *layer 1* are 1-hop downstream, while the genes in *layer 2* are 1- and 2-hops downstream, and so on. We then measured the degree of overlap between the key driver perturbation signature and the N-hop downstream nodes relative to that key driver for each layer using Fisher’s Exact Test. In order to control for the number of downstream neighborhood layers tested for each perturbation signature, we used the Benjamini-Hochberg FDR adjustment method on the enrichment p-values from each gene’s perturbation signature in all of the corresponding layers tested (n = 10).

**Supplementary Figures**

**
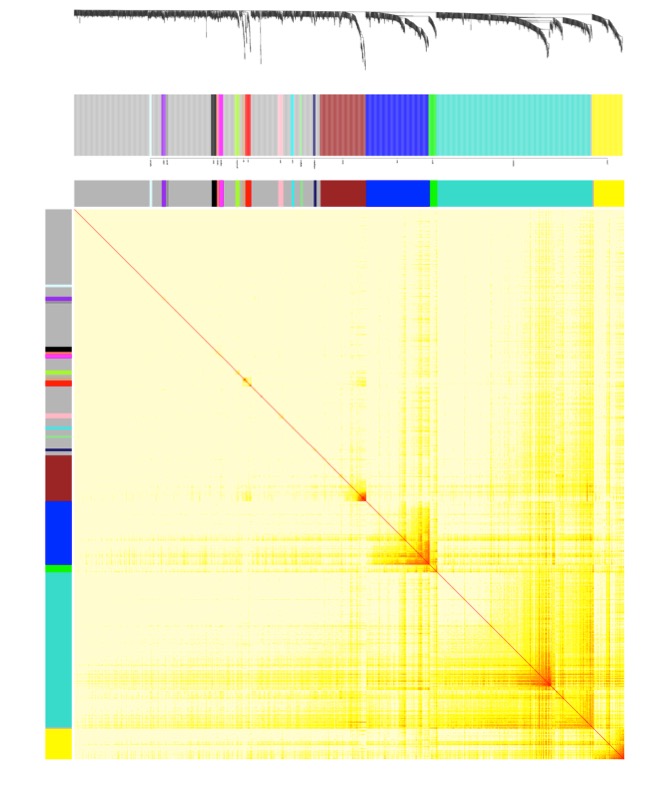
**

**Supplementary Figure 1**. The topological overlap matrix plot of the protein co-expression network constructed from the proteomics data from the autopsied brains in the MSBB cohort, along with the dendrogram showing the tree cutting process used to define modules (above).


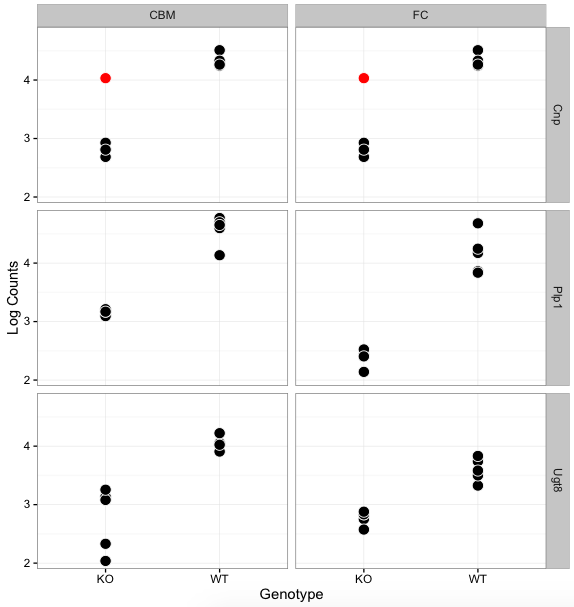


**Supplementary Figure 2**. Confirmation that the key driver knockouts abrogate gene expression of the key driver in the RNA-seq experiments. For each of the key driver knockouts whose genome-wide gene expression was profiled in this study using RNA-seq, we plotted the log_10_ counts overlapping that gene in both the wildtype (WT) and knockout (KO) samples. Notably, one of the matched samples from *Cnp* was detected as an outlier in both the CBM and FC brain regions (red), due to suspected mislabeling. These samples were removed prior to downstream differential expression analysis.

**References**

1. Zhang B, Gaiteri C, Bodea L-G, Wang Z, McElwee J, Podtelezhnikov AA, Zhang C, Xie T, Tran L, Dobrin R *et al*: **Integrated Systems Approach Identifies Genetic Nodes and Networks in Late-Onset Alzheimer’s Disease**. *Cell* 2013, **153**:707-720.

2. Zhang B, Horvath S: **A General Framework for Weighted Gene Co-Expression Network Analysis**. *Statistical Applications in Genetics and Molecular Biology*, **4**.

3. Zhu J, Wiener MC, Zhang C, Fridman A, Minch E, Lum PY, Sachs JR, Schadt EE: **Increasing the Power to Detect Causal Associations by Combining Genotypic and Expression Data in Segregating Populations**. *PLoS Comput Biol* 2007, **3**(4):e69.

4. Zhu J, Zhang B, Smith EN, Drees B, Brem RB, Kruglyak L, Bumgarner RE, Schadt EE: **Integrating large-scale functional genomic data to dissect the complexity of yeast regulatory networks**. *Nature Genetics* 2008, **40**:854-861.

5. Zhang Y, Chen K, Sloan SA, Bennett ML, Scholze AR, O'Keeffe S, Phatnani HP, Guarnieri P, Caneda C, Ruderisch N *et al*: **An RNA-Sequencing Transcriptome and Splicing Database of Glia, Neurons, and Vascular Cells of the Cerebral Cortex.** *The Journal of neuroscience : the official journal of the Society for Neuroscience* 2014, **34**:11929-11947.

6. Mishra A, Macgregor S: **VEGAS2: Software for More Flexible Gene-Based Testing.** *Twin research and human genetics : the official journal of the International Society for Twin Studies* 2015, **18**:86-91.

7. Seyfried NT, Dammer EB, Swarup V, Nandakumar D, Duong DM, Yin L, Deng Q, Nguyen T, Hales CM, Wingo T *et al*: **A Multi-network Approach Identifies Protein-Specific Co-expression in Asymptomatic and Symptomatic Alzheimer's Disease**. *Cell Syst* 2017, **4**(1):60-72 e64.

8. Cox J, Neuhauser N, Michalski A, Scheltema RA, Olsen JV, Mann M: **Andromeda: a peptide search engine integrated into the MaxQuant environment.** *Journal of proteome research* 2011, **10**:1794-1805.

9. Cox J, Hein MY, Luber CA, Paron I, Nagaraj N, Mann M: **Accurate proteome-wide label-free quantification by delayed normalization and maximal peptide ratio extraction, termed MaxLFQ.** *Molecular & cellular proteomics : MCP* 2014, **13**:2513-2526.

10. Luber CA, Cox J, Lauterbach H, Fancke B, Selbach M, Tschopp J, Akira S, Wiegand M, Hochrein H, O'Keeffe M *et al*: **Quantitative proteomics reveals subset-specific viral recognition in dendritic cells.** *Immunity* 2010, **32**:279-289.

11. Emery B, Agalliu D, Cahoy JD, Watkins TA, Dugas JC, Mulinyawe SB, Ibrahim A, Ligon KL, Rowitch DH, Barres BA: **Myelin Gene Regulatory Factor Is a Critical Transcriptional Regulator Required for CNS Myelination**. *Cell* 2009, **138**:172-185.

12. Storey JD, Tibshirani R: **Statistical significance for genomewide studies.** *Proceedings of the National Academy of Sciences of the United States of America* 2003, **100**:9440-9445.

13. Storey J: **qvalue: Q-value estimation for false discovery rate control.**; 2015.

14. Benjamini Y, Hochberg Y: **Controlling the False Discovery Rate: A Practical and Powerful Approach to Multiple Testing**. *Journal of the Royal Statistical Society Series B (Methodological)* 1995, **57**:289 - 300.
